# New Dark Area Sensitive Tone Mapping for Deep Learning Based Traffic Sign Recognition

**DOI:** 10.3390/s18113776

**Published:** 2018-11-05

**Authors:** Jameel Ahmed Khan, Donghoon Yeo, Hyunchul Shin

**Affiliations:** Division of Electrical Engineering, Hanyang University ERICA, Ansan 426-791, Korea; dhyeo@hanyang.ac.kr

**Keywords:** Korean Traffic Sign Detection, Dark Area Sensitive Tone Mapping (DASTM), classical tone mapping, luminance enhancement

## Abstract

In this paper, we propose a new Intelligent Traffic Sign Recognition (ITSR) system with illumination preprocessing capability. Our proposed Dark Area Sensitive Tone Mapping (DASTM) technique can enhance the illumination of only dark regions of an image with little impact on bright regions. We used this technique as a pre-processing module for our new traffic sign recognition system. We combined DASTM with a TS detector, an optimized version of YOLOv3 for the detection of three classes of traffic signs. We trained ITSR on a dataset of Korean traffic signs with prohibitory, mandatory, and danger classes. We achieved Mean Average Precision (MAP) value of 90.07% (previous best result was 86.61%) on challenging Korean Traffic Sign Detection (KTSD) dataset and 100% on German Traffic Sign Detection Benchmark (GTSDB). Result comparisons of ITSR with latest D-Patches, TS detector, and YOLOv3 show that our new ITSR significantly outperforms in recognition performance.

## 1. Introduction

Development of automatic traffic sign recognition systems with high accuracy is a very important issue because this system can alert the driver about the road conditions and speed limits by recognizing the traffic signs from a large distance. Recognizing traffic signs from a road image is a challenging task due to occlusions, variations in illumination, variable speed of vehicle, and variation in size of a traffic sign due to variable distance. Illumination of the scene has also high impact on the visibility of objects in the image. We observed that traffic signs often appear at the top portion of road images and the bright region of sky in the background can seriously affect the visibility of these traffic signs. Low illumination on a traffic sign region due to bright background affects the recognition process and detector may fail to detect these traffic signs. Several luminance enhancement techniques have been proposed in the last few years, such as global tone mapping by Erik et al. [1] and global and local tone mapping by Shin et al. [2]. However, these techniques are not effective for traffic sign recognition.

Korean Traffic Sign Detection (KTSD) dataset by Yawar et al. [3] is a challenging dataset because it contains traffic signs with low visibility due to low illumination and small size. Yawar et al. trained their D-patches [3] on three super classes, Prohibitory class, Mandatory class, and Danger class, and achieved an average detection accuracy of 72.37% on KTSD. However, there were some errors in the ground truth annotations of KTSD and we corrected those errors and tested it on D-patches. After correcting the erroneous annotations, detection accuracy of D-patches increased to 79.6% on KTSD. We trained YOLOv3 by Joseph et al. [4] on our self-made dataset and tested it on KTSD. It gave us recognition rate of 73.9%. Recognition rate of the recent TS detector on KTSD is 86.6% [5].

The goal of our research was to increase the detection accuracy on KTSD dataset, and hence we developed a new Intelligent Traffic Sign Recognition (ITSR) system with illumination preprocessing capability. ITSR is an intelligent system that takes the input image and processes it to detect traffic signs of prohibitory, mandatory, and danger classes, even in low illumination condition. This system is fast and efficient as compared to other detection methods and the detection accuracy of ITSR is 90.07% on KTSD. We also tested ITSR on German Traffic Sign Benchmark (GTSDB) [6] and achieved 100% accuracy. For developing ITSR, the following approach was used.

We made a new training dataset of same three super classes with large, medium, and small size traffic signs. This dataset contains 3300 images on various road conditions in South Korea.

**Figure 1:**
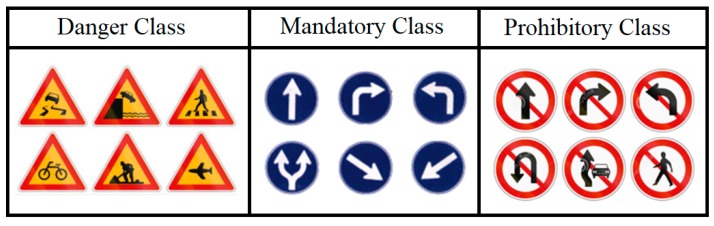
Three super classes of traffic signs used for experiment.

In Figure 1, examples of three super classes of Korean traffic signs have been shown.

**Figure 2:**
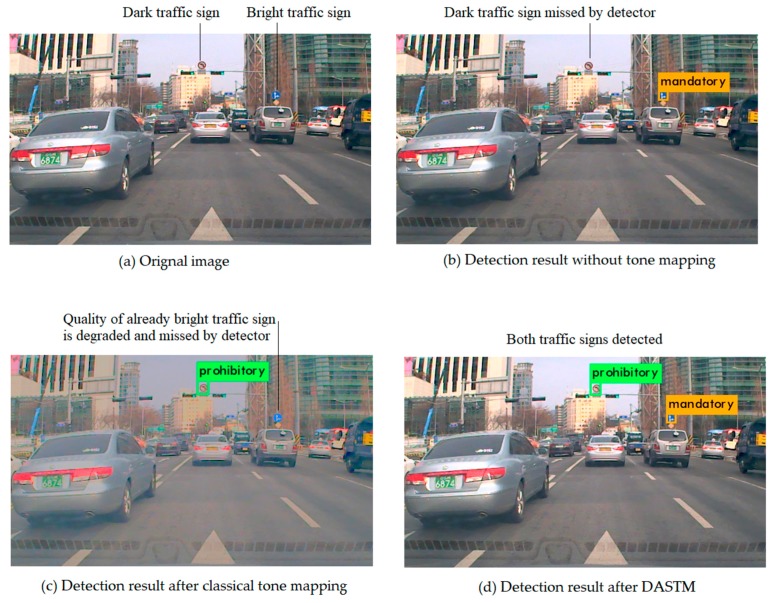
Typical results of detection: (**a**) original image; (**b**) detection without tone mapping, in which a dark traffic sign is missed; (**c**) detection after classical tone mapping, in which a bright traffic sign is missed; and (**d**) detection after DASTM, in which both traffic signs are successfully detected.

For the detection of traffic signs, we have used TS detector and trained it on our new dataset. TS detector is an optimized version of YOLOv3 [4]. For detecting small traffic signs, TS detector down sampled the input image by the factor of 32, 16 and 4 to make the grid denser and pre-calculate the anchor box size from training data. Instead of using three anchor boxes at each detection stage, TS detector uses five anchor boxes in the first detection stage and two anchor boxes in the second and the third detection stage [5]. We analyzed all error cases and concluded that TS detector is failing to detect those traffic signs that have low illumination. To resolve this problem and to enhance the illumination of dark traffic signs, we used classical tone mapping technique. We applied this technique on test dataset and analyzed the results. Although the luminance of dark traffic signs enhanced, this technique degraded the quality of already bright traffic signs, and detection algorithm failed to show satisfactory performance. To overcome this problem, we developed our new Dark Area Sensitive Tone Mapping (DASTM) algorithm. By using DASTM, we can enhance the luminance of only dark regions in images, and thus we can achieve significant improvement in detection accuracy on KTSD. In Figure 2, typical detection results, without tone mapping, after classical tone mapping, and after DASTM, are compared.

## 2. Related Works

### 2.1. Traffic Sign Detection

In recent years, several techniques for traffic sign detection have been proposed. Yawar et al. in D-patches [3] used discriminative patch approach to detect occluded traffic signs. D-patches is capable of occlusion handling in detection process. They used ACF detection [7] framework and extracted features from discriminative patches of the traffic signs. This approach performed well in occlusion cases but it failed to detect low illumination traffic signs. They tested D-Patches on KTSD and German traffic sign detection benchmark (GTSDB), and achieved 72.37% accuracy on KTSD and 100% accuracy on GTSDB. Zhe et al. [8] collected 100,000 images to make traffic sign benchmark in China and named this benchmark Tsinghua-Tencent 100K. Zhe et al. used convolutional neural network (CNN) to detect and classify the traffic signs. Chung et al. [9] used You Look Only Once (YOLO) framework for detection and trained it on Belgium Traffic Sign Dataset [10]. They used 13 classes of different shapes and colors. They achieved 33.8% Mean Average Precision (MAP) on their test dataset. Marcin et al. proposed a system to read speed limit of traffic signs by RANSAC method [11]. They only proposed the method of reading the speed limit, but fast and accurate detection of traffic sign is still an important issue. Ayoub et al. proposed border’s color and shape features classification by random forests to detect traffic signs [12]. Amara et al. used deep learning algorithm to detect traffic signs [13]. They achieved 97.6% recognition rate on GTSDB. Chunsheng et al. [14] proposed a Traffic Sign Recognition (TSR) framework that extracts region of interest before detection. They used split-flow cascade tree detector and rapid occlusion-robust traffic sign classification method for detecting traffic signs. Zhonrong et al. [15] used Faster R-CNN [16] for traffic sign detection and achieved mAP result of 0.34493. Similarly, some recent detection techniques of CNN can also be used for traffic signs detection from videos, such as “Two-Stream Multirate Recurrent Neural Network” proposed by Zhiqiang et al. [17]. In [18], authors compared the classification accuracy with standard deviation.

### 2.2. Detection Methods

Joseph et al. proposed YOLO 9000, a state of the art, real time object detection system [19]. This system can detect wide variety of object classes. YOLO 9000 can detect 9000 objects in real time. SSD model [20] uses single deep neural network for object detection while YOLOv3 [4] is updated version of YOLOv2 [19]. YOLOv3 uses logistic regression for calculating the confidence score of object in each bounding box. YOLOv3 uses variant of Darknet, which has a 53-layer network trained on Imagenet dataset [21]. However, 53 more layers are stacked on it to form a 106-layer fully convolutional architecture for detection tasks. YOLOv3 includes many important elements like residual blocks, skip connections, and upsampling in its architecture.

### 2.3. Tone Mapping

Erik et al. presented a technique named “Photographic Tone Reproduction for Digital Images” [1]. In this method, initially log average luminance is calculated and then the luminance is enhanced by simple tone mapping as given below.

(1) Ld(x, y) = L(x,y)/[1+L(x,y)] 

where *Ld*(*x*, *y*) is enhanced luminance and *L*(*x*, *y*) is initial luminance.

Shin et al. [2] proposed a modified mapping function that considers the block level log-average and the log-average of the whole image. A parameter α is used to set the tradeoff between the global average and the local average. When α = 0, only global tone mapping is performed; when α = 1, only block level tone mapping is performed; and 0 < α < 1 is for tradeoff between global and block level tone mapping. The input image *I*(*x*, *y*) can be obtained from the luminance component *L*, and reflectance component *R* of an image. Now, the global log-average luminance is calculated by the following formula.

(2) I(x, y) = L (x, y) × R (x, y) 

(3) $$\;Lavg=\exp(\frac{1}{N}\overset{\;}{\underset{x,y}{\sum }}\log\left[\delta +L\left(x,y\right)\right])\;$$

where *N* is total number of pixels in an image, and *δ* is a small value to avoid singularity.

Drago et al. [22] proposed a technique called “adaptive logarithmic mapping” for producing tuned images with high dynamic contents. They proposed gamma correction procedure for improving the contrast of dark areas of image.

## 3. Proposed Intelligent Traffic Sign Recognition (ITSR) System

Our Intelligent Traffic Sign Recognition (ITSR) system can detect and classify three super classes of traffic signs simultaneously. This system consists of two processing modules, tone mapping module and detection module. In tone mapping module, we have developed our new Dark Area Sensitive Tone Mapping (DASTM) technology. In DASTM, we have divided the input image into two regions: dark region, and bright region, using a luminance threshold. DASTM is the first intelligent approach that performs multiple luminance range based tone mapping on the input image. For detection module, we used TS detector [5], our optimized version of YOLOv3 [4]. YOLOv3 is a deep learning detection and classification algorithm based on Darknet. YOLOv3 uses filtering in convolution layers to resize the image into small grids and detection is performed in three detection stages. YOLOv3 uses three anchor boxes at each detection stage and the average loss is calculated in each iteration. While TS detector [5] pre-calculates the size of anchor boxes from training data and uses five anchor boxes in the first detection stage, and two anchor boxes in the remaining two detection stages. TS detector resizes the image into a denser grids, suitable for detecting small objects [5]. For training, we have developed our new dataset on Korean traffic signs with prohibitory, mandatory and danger classes. We tested our system on KTSD [3] and GTSBD [6]. For evaluation of detected traffic signs, we used Mean Average Precision (MAP) and achieved 90.07% on KTSD and 100% on GTSDB.

### 3.1. Failure of Classical Tone Mapping and Need of DASTM

**Figure 3:**
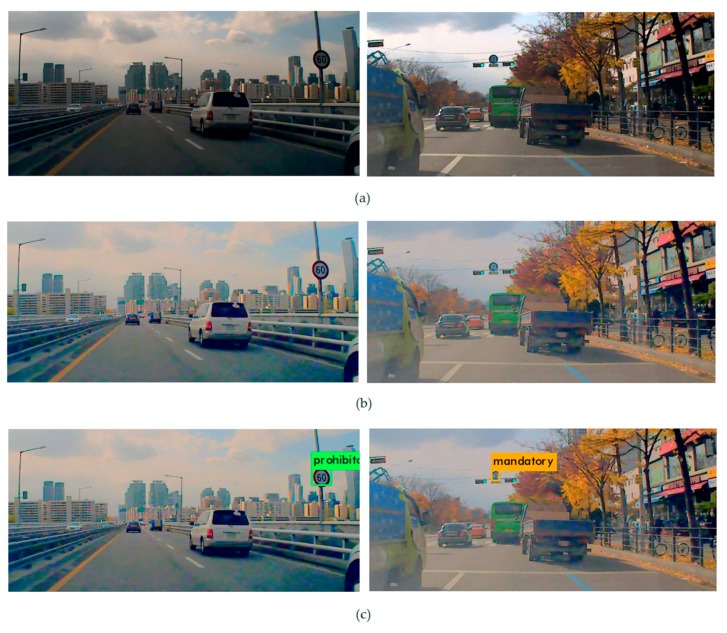
(**a**) Original dark images; (**b**) improved luminance by tone mapping; and (**c**) detection of traffic signs.

Traffic signs generally appear at the top region of an image, and usually the image of a traffic sign becomes dark, due to the bright background sky region, as shown in Figure 3.

The detector usually misses these dark traffic signs and detection accuracy is affected by this problem. To detect these traffic signs, it is necessary to enhance their luminance and make them clear. To resolve this problem, one may calculate the luminance of input image and apply a classical tone mapping technique. The equation of classical tone mapping is given below.

(4)  Nlum = Slum×(1+C)/(Slum+C) 

where *Slum* is the initial scaled luminance of the input image and its value is 0 ≤ *Slum* ≤ 1. The *Nlum* is new calculated scaled luminance after tone mapping and *C* is the tone mapping parameter for luminance enhancement. Although dark traffic signs became bright by this global tone mapping technique, the quality of already bright traffic signs was degraded. This is because the sensitivity of bright region is decreased, while the sensitivity of dark region is increased. Therefore, it is necessary to develop a new technology that can enhance the sensitivity of only dark regions without affecting bright regions too much.

### 3.2. Dark Area Sensitive Tone Mapping (DASTM)

In this research, we have developed a new tone mapping method, DASTM, in which the luminance range is divided into multiple regions, and different tone mapping functions are used for the divided regions. For traffic sign detection, we have divided this range into two regions, dark region and bright region. The regions are separated by setting a threshold value. We can further divide this range into more regions depending upon applications.

First, we calculate the luminance of input image, scale it from zero to one, and name it as “*Slum*”. We set a threshold value “*thr*” to divide the range of scaled luminance. The region with *Slum* below “*thr*” is the dark region and the region with *Slum* above “*thr*” is the bright region. In DASTM, *Nlum* values for dark and bright regions are calculated separately. Let *Slum* be scaled luminance (0 ≤ *Slum* ≤ 1), *Nlum* be new scaled luminance, *thr* be *threshold* value, and *C* be a parameter, then *Nlum* values for both the regions are computed by using the following equations.

If *Slum* ≤ *thr*

*Nlum* = *Slum* × (1 + *C*)/(*Slum* + *C*) (5)

else

*Nlum* = *m* × *Slum* + *b* (6)

Equation (6) is a straight line equation in which *m* is the slope of bright region line and *b* is a constant. The slope *m* of a line passing two points, (*x*1, *y*1) and (*x*2, *y*2), is given by

*Slope* = (*y*2 − *y*1)/(*x*2 − *x*1) (7)

**Figure 4:**
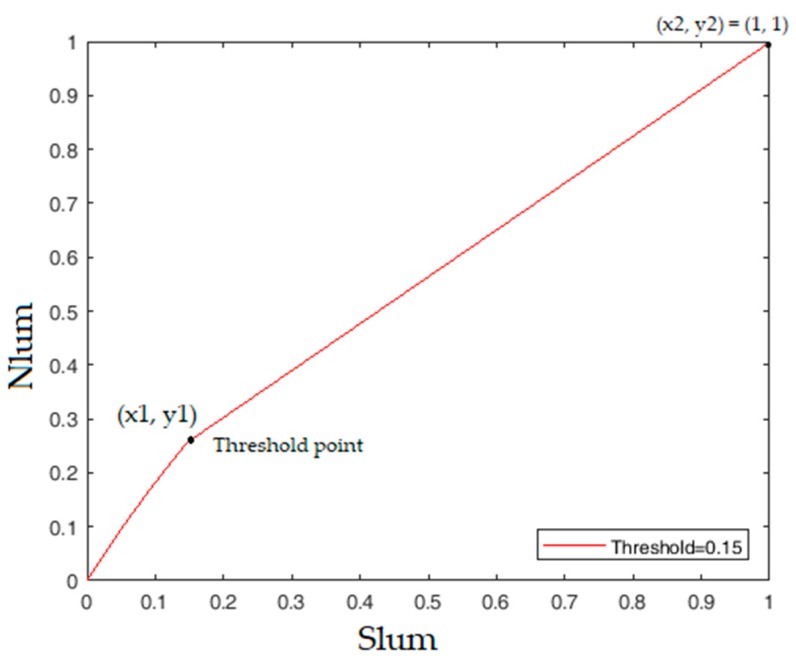
Behavior of *Nlum* above and below threshold point.

Let (*x*1, *y*1) = (*Slum*, *Nlum*) be the point when *Slum* = *thr*. We calculated *Nlum* at that point using Equation (5), and (*x2*, *y2*) = (1, 1) is the ending point of the line, as shown in Figure 4. After calculating *m*, we used the point (*Slum*, *Nlum*) = (1, 1) to find the value of *b* using Equation (6).

**Figure 5:**
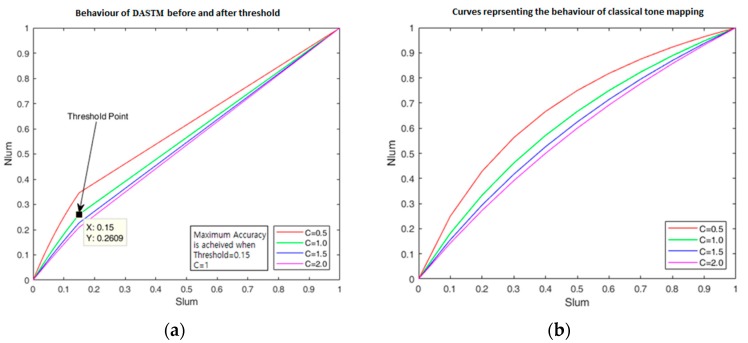
Graphical representation of new-scaled luminance: (**a**) new DASTM; and (**b**) classical tone mapping.

Figure 5 shows the behavior of *Nlum* enhancement by changing the values of *C* from 0.5 to 2.0. The maximum MAP is achieved when *C* = 1 and *thr* = 0.15 in DASTM. Further analysis of MAP is given using tables in the Section 5. Although there is a slight change in luminance in bright region, that change is negligible.

## 4. Experimental Results

### 4.1. Making New Training Dataset

We have made our new dataset of Korean traffic signs for training. There are 3300 images of roads in Korea with traffic signs of various sizes. We have annotated these images manually and used the dataset to train our detection module.

### 4.2. Training and Testing

**Figure 6:**
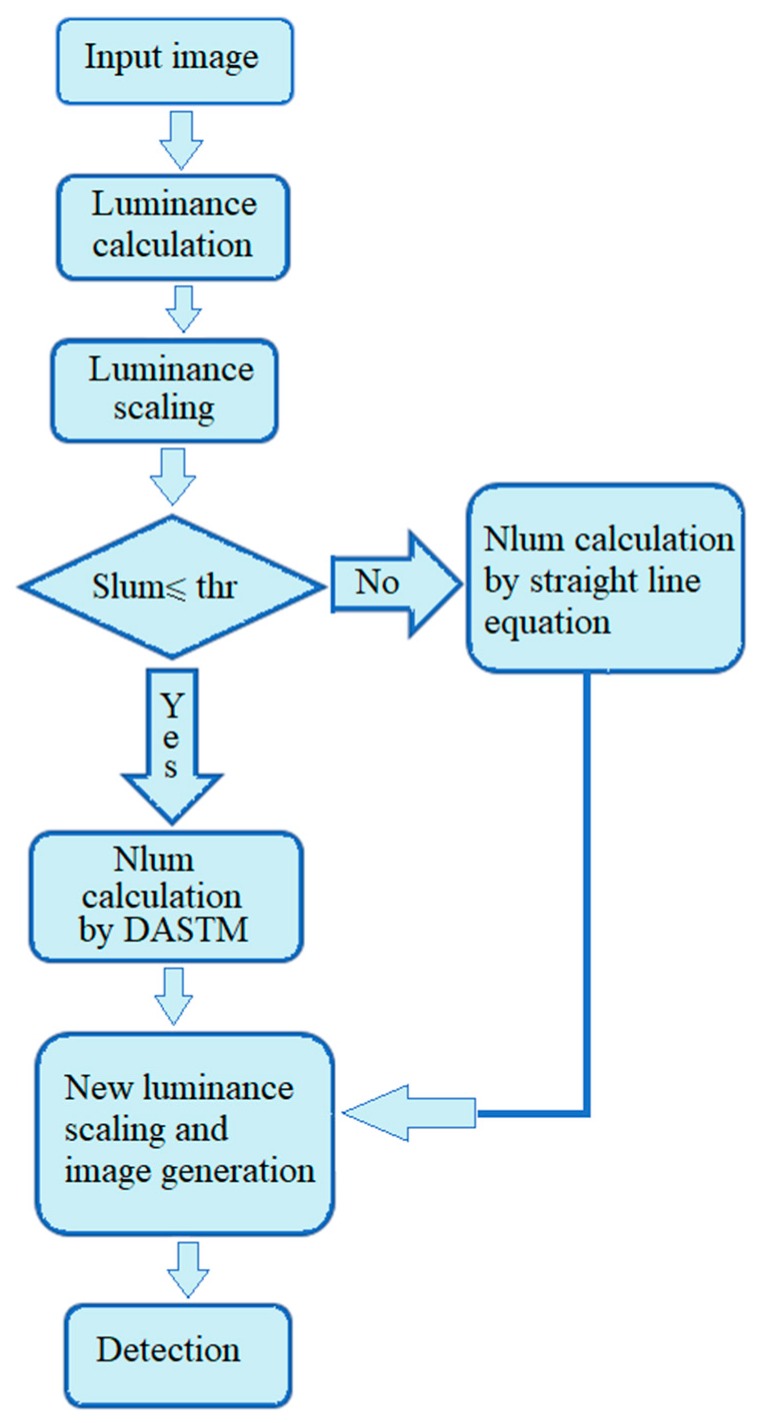
Flow diagram of the new Intelligent Traffic Sign Recognition System.

We have combined DASTM with TS detector, the optimized version of YOLOv3 framework to detect small size and low illumination traffic signs well. For our experiments, we used a computer containing core i7 CPU, 16GB RAM, under Linux operating system. We also installed NVIDIA TITAN X GEFORCE GTX GPU on it. After using the initial weights from ImageNet [21] dataset, fine-tuning of the detection module has been done using our new dataset. The training process took four to five days by using a single GPU board, with NVIDIA CUDA [23] as a parallel computing platform. The data were moved from CPU main memory to GPU memory and then to the cores of GPU for parallel execution. While CPU instructs the GPU for processing of data, all the computation was done on the GPU. Execution results were moved back to GPU memory and then to main memory. We continued training until the average loss was reduced. Validation data were used for training analysis to avoid overfitting. Then, the weight file and configuration file were saved to be used during testing. The DASTM was applied only on testing data. The detection results were compared with the ground truth to calculate the MAP for evaluation. Figure 6 shows the overall flow of our algorithm. By using DASTM, the sensitivity of dark region is enhanced and the detection accuracy is significantly increased.

### 4.3. Evaluation Method

The Mean Average Precision (MAP) was adopted to analyze the recognition rates. The detected bounding box was compared with a ground truth bounding box to decide whether the prediction is true. The Intersection Over Union (IOU) of bounding boxes was used to decide if the prediction is true positive. If IOU ≥ 40%, the prediction is classified as a true positive. Otherwise, the prediction is false positive. The IOU is calculated as follows.

IOU = (Bounding box area of intersection)/(Bounding box area of union)(8)

Then, the Precision and Recall values were computed by using

*Precision* = *TP*/(*TP* + *FP*) (9)

**Figure 7:**
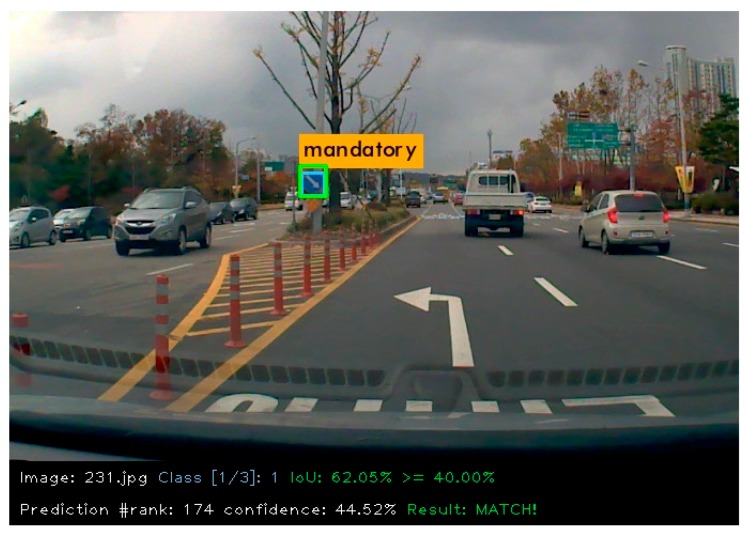
An IOU calculation example.

*Recall* = *TP*/(*TP* + *FN*) (10)

where *TP* is True Positive, *FP* is False Positive, and *FN* is False Negative. The Precision–Recall curves were drawn using confidence score of prediction, and the area under the curve was computed to get Average Precision. The MAP calculation codes form Cartucho [24] were used to analyze the results. Figure 7 shows an example of a mandatory class prediction from KTSD, in which detected bounding box is green, ground truth bounding box is sky blue, and for this example IOU is 62.05%.

**Figure 8:**
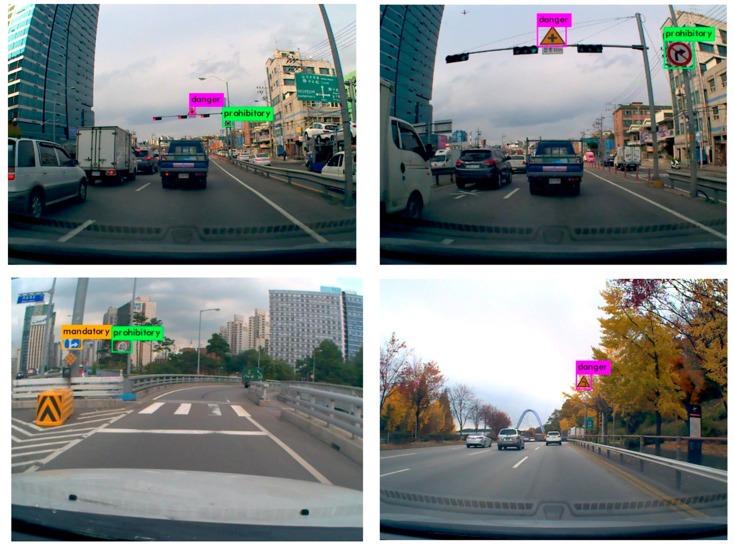
Detection examples on Korean Traffic Sign Dataset.

Figure 8 shows experimental results of traffic sign detection on KTSD. We can see that ITSR is able to detect small traffic signs from large distances. ITSR can detect all three classes simultaneously, while D-patches method detects one class at a time.

## 5. Discussion on Experimental Results

**Figure 9:**
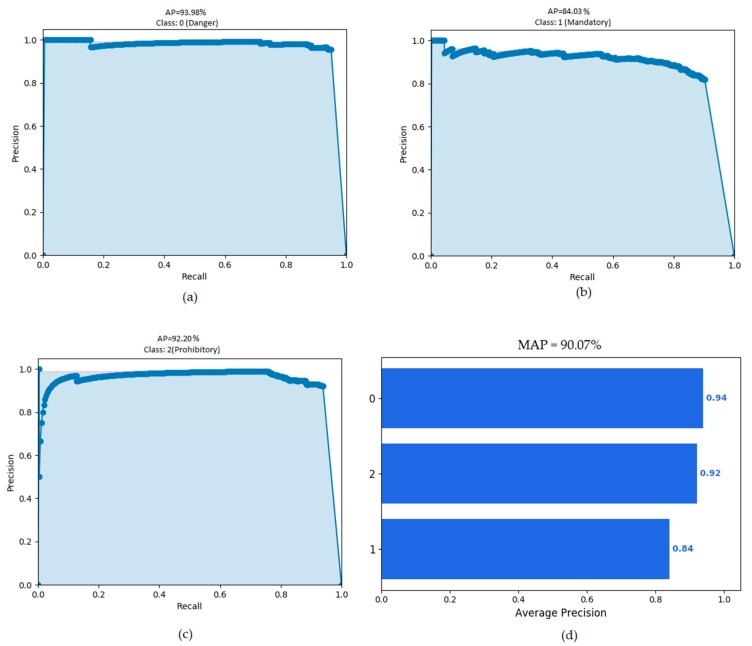
Best results achieved by ITSR on Korean Traffic Sign Dataset for three different classes: (**a**) average precision of danger class; (**b**) average precision of mandatory class; (**c**) average precision of prohibitory class; and (**d**) mean average precision of all three classes.

ITSR gives 90.07% MAP on KTSD and 100% on GTSBD. Further detailed results are given in Table 1, Table 2, Table 3, Table 4 and Table 5. Figure 9 shows the graphs of recognition rates on KTSD. In Figure 9a–c, area under the curve (in sky blue shade) is measured for calculation of Average Precision (AP) of individual class. We can see that AP of danger class is 93.98%, and this value is maximum among all three classes. MAP is the mean of average precisions of all three classes as shown in Figure 9d.

In Table 1, detection results are compared after applying DASTM and classical tone mapping on KTSD and GTSDB. This comparison shows that our proposed DASTM gives best detection performance on both datasets and shows significantly higher recognition rates.

In Table 2, mean values of average precisions (MAP) of three classes with their standard deviation (±STD) are compared. This comparison also shows that ITSR gives best detection performance on both KTSD and GTSDB datasets.

ITSR uses DASTM as pre-processing before detection, so it is slower than TS detector. DASTM takes 0.0754 s to process one frame of 800 × 600 resolution. Although DASTM makes the detector slower, this technique increases the recognition rate and makes the system reliable. Comparison of processing times of ITRS with other methods is shown in Table 3.

DASTM is an effective, reliable, and excellent illumination pre-processing technique for the intelligent traffic sign recognition system. DASTM is a very useful technique that increases the detection sensitivity of only dark regions of an image. In Table 4 and Table 5, for different values of *C*, recognition rate is computed after applying DASTM and classical tone mapping. Table 4 shows the detection performance of DASTM, while Table 5 shows the detection performance of classical tone mapping. During experiment on DASTM, for a specific value of *C*, maximum recognition rate is achieved by changing *thr*. Table 4 shows the reliability of ITSR when *C* = 0.5, *C* = 1, *C* = 1.5 and *C* = 2. The behavior of *Nlum* by changing *C* is also shown in Figure 5. By applying DASTM on KTSD, maximum MAP of 90.07% is achieved at *C* = 1 and *thr* = 0.15, and, by applying classical tone mapping, maximum MAP of 83.35% is achieved at *C* = 1.5. The main reason for bad performance after classical tone mapping is the decreased sensitivity of already bright traffic signs. MAP comparison of both techniques shows that DASTM is outperforming classical tone mapping and detection accuracy of DASTM is significantly better.

### Possible Future Research

DASTM divides the luminance range into two regions; however, the range can be divided into multiple regions. For each region, different tone mapping function can be applied, depending upon the need of the system. We used DASTM as pre-processing for traffic sign recognition. This technique can be applied for the recognition of other dark objects in the scenery. We plan to do research to develop effective tone mapping techniques for various vision applications.

## 6. Conclusions

MAP results show that ITSR gives the best performance on challenging KTSD and GTSDB. It is an effective method for detecting low illumination and small-sized traffic signs. Although ITSR is slightly slower than TS detector, this system is an efficient and reliable system, and its performance is significantly better than those of other detectors. We used three classes of traffic signs in our experiment; however, it is possible to train ITSR on more than three classes. DASTM module makes ITSR efficient by intelligent luminance pre-processing to increase sensitivity in dark regions. By using linear mapping, although there is a slight change in luminance of bright region, the change is negligible and does not affect the performance of the detector. We can also use DASTM as a pre-processing module for other applications. In DASTM, we divided the luminance range into two regions, but the range can be divided into multiple regions if necessary, depending upon its application areas.

## Figures and Tables

**Table 1:** Comparison of DASTM with classical tone mapping.

Tone Mapping Method	Dataset	Maximum MAP
DASTM	KTSD	90.07%
Classical	KTSD	83.35%
DASTM	GTSBD	100%
Classical	GTSBD	95.26%

**Table 2:** Comparison of ITSR with D-Patches, YOLOv3 and TS detector.

Detection Method	MAP on KTSD ± STD	MAP on GTSBD ± STD
ITSR (DASTM)	90.07% ± 4.33	100% ± 0
D-Patches	79.60% ± 5.82	100% ± 0
YOLOv3	73.94% ± 6.12	96.53% ± 2.31
TS detector	86.61 ± 5.33	97.82% ± 1.91

**Table 3:** CPU time comparison of ITSR with D-Patches, YOLOv3 and TS detector.

Detection Method	Resolution	Time to Process 1 Frame
ITSR (DASTM)	800 × 600	0.134 s (on GPU)
D-Patches	800 × 600	2.2 s (on CPU)
YOLOv3	800 × 600	0.050 s (on GPU)
TS detector	800 × 600	0.059 s (on GPU)

**Table 4:** MAP analysis after applying DASTM on KTSD for different *C* and *thr*.

*C*	Threshold	*Nlum* at Point *Slum* = *thr*	MAP
0.5	0.05	0.13	82.40%
0.5	0.10	0.25	89.71%
0.5	0.15	0.34	84.50%
0.5	0.20	0.42	83.33%
1	0.10	0.18	86.16%
1	0.15	0.26	90.07%
1	0.20	0.33	84.50%
1.5	0.10	0.15	84.50%
1.5	0.15	0.22	87.50%
1.5	0.20	0.29	87.20%
2	0.10	0.14	84.01%
2	0.15	0.20	86.02%
2	0.20	0.27	88.06%
2	0.25	0.33	84.50%

**Table 5:** Results of MAP using classical tone mapping on KTSD for different values of *C*.

*C*	MAP
0.5	82.91%
1.0	83.20%
1.5	83.35%
2.0	82.88%
